# Attentional and visual demands for sprint performance in non-fatigued and fatigued conditions: reliability of a repeated sprint test

**DOI:** 10.1186/1471-2474-11-84

**Published:** 2010-05-04

**Authors:** Inge HF Reininga, Koen APM Lemmink, Ron L Diercks, Arina T Buizer, Martin Stevens

**Affiliations:** 1 Department of Orthopaedics, University Medical Center Groningen, University of Groningen, Hanzeplein 1, PO Box 30.001, 9700 RB Groningen, The Netherlands; 2 University Center for Sport, Exercise and Health, University Medical Center Groningen, University of Groningen, Groningen, The Netherlands; 3 Center for Human Movement Sciences, University Medical Center Groningen, University of Groningen, Groningen, The Netherlands; 4 School of Sports Studies, Hanze University of Applied Sciences Groningen, Groningen, the Netherlands; 5 Department of Sports Medicine, University Medical Center Groningen, University of Groningen, Groningen, The Netherlands

## Abstract

**Background:**

Physical performance measures are widely used to assess physical function, providing information about physiological and biomechanical aspects of motor performance. However they do not provide insight into the attentional and visual demands for motor performance. A figure-of-eight sprint test was therefore developed to measure the attentional and visual demands for repeated-sprint performance. The aims of the study were: 1) to assess test-retest reliability of the figure-of-eight sprint test, and 2) to study the attentional and visual demands for sprint performance in a non-fatigued and fatigued condition.

**Methods:**

Twenty-seven healthy athletes were included in the study. To determine test-retest reliability, a subgroup of 19 athletes performed the figure-of-eight sprint test twice. The figure-of-eight sprint test consisted of nine 30-second sprints. The sprint test consisted of three test parts: sprinting without any restriction, with an attention-demanding task, and with restricted vision. Increases in sprint times with the attention-demanding task or restricted vision are reflective of the attentional and visual demands for sprinting. Intraclass correlation coefficients (ICCs) and mean difference between test and retest with 95% confidence limits (CL) were used to assess test-retest reliability. Repeated-measures ANOVA were used for comparisons between the sprint times and fatigue measurements of the test parts in both a non-fatigued and fatigued condition.

**Results:**

The figure-of-eight sprint test showed good test-retest reliability, with ICCs ranging from 0.75 to 0.94 (95% CL: 0.40-0.98). Zero lay within the 95% CL of the mean differences, indicating that no bias existed between sprint performance at test and retest. Sprint times during the test parts with attention-demanding task (P = 0.01) and restricted vision (P < 0.001) increased significantly compared to the base measurement. Furthermore the sprint times and fatigue measurements increased significantly in fatigued condition. There was a significant interaction effect between test part and level of fatigue (P = 0.03).

**Conclusions:**

High ICCs and the absence of systematic variation indicate good test-retest reliability of the figure-of-eight sprint test. The attentional and visual demands for sprint performance, in both a non-fatigued and fatigued condition, can be measured in healthy team-sport athletes with the figure-of-eight sprint test.

## Background

Physical performance measures are widely used to assess physical function for sports or rehabilitation purposes. Although such measures for e.g. sprint performance, jump strength and hop performance may deliver relevant information about a number of physiological and biomechanical variables, they do not provide insight into the role of sensorimotor control in motor performance. Athletes use several sources of information, such as proprioceptive and visual information, required to perform motor tasks. Sensorimotor control demands athletes' attentional involvement. The more skilled an athlete is in performing a certain motor task, the less attentional involvement in the task is needed, i.e. the performance has become fairly automatic [1]. Furthermore, peripheral vision is an important visual information source that provides valuable information for the control of the athlete's own movements [1].

Dependency on attention can be measured by adding a concurrent attention-demanding task to a primary motor task. The level of (dual-task) interference serves as an estimate of the amount of attention needed to perform the primary motor task. Indeed, when the motor task is performed worse if executed simultaneously with the attention-demanding task it is argued that performance of the motor task is not yet automated [2,3]. Dependency on visual information can be measured by restricting the available visual information while performing the primary motor task. In this case, decrease in motor performance is reflective of the dependency on visual information [3].

The ability to perform sports activities under fatigued conditions is of great importance. Sports injuries tend to occur at the end of a sporting event, when the athlete is fatigued [4,5]. Fatigue is caused by a combination of different physiological mechanisms occurring at both the central and the peripheral levels, leading to decreased sensorimotor control [6]. Some studies have reported decreased lower-limb joint proprioception [5,7], increased joint laxity and a delay in muscle response [7,8] following fatiguing exercise. Moreover, research has proven that dependency on attention and visual information for sensorimotor control may increase with fatigue [9,10].

In field-based team sports, half of all injuries affect the lower extremities [11]. Research has established that damage to the musculoskeletal system of the lower extremities results in loss of proprioceptive information [12,13]. Rehabilitation after these injuries may be seen as sensorimotor learning, where the injured athlete has to relearn coordinated movement patterns based on an altered proprioceptive feedback. During this learning process, the attentional demands for sensorimotor control are raised [2,3]. Furthermore, until the motor programs are adapted to the altered proprioceptive feedback, the athlete has to depend on other sources of information. This results, for example, in a disproportional dependency on vision as an important information source to perform motor tasks without performance loss [3]. Hence the hypothesis is that, while an athlete recovering from a lower-limb injury is disproportionately dependent on attention and visual information while performing physical activities, particularly in a fatigued physical condition, he is not yet fully recovered and a return to strenuous physical activities such as sports may entail an increased risk of reinjury.

Based on the aforementioned thoughts, it can be argued that a test is needed which provides insight not only into the physical condition but also into the attentional and visual demands for sensorimotor control. A figure-of-eight sprint test of an intermittent and multidirectional nature was developed, reflecting two basic characteristics of field-based team sports. To determine the attentional and visual demands for sprinting, the sprint test has to be performed with a concurrent attention-demanding task or with restricted vision. Within this framework the protocol of sprinting and recovery was developed with two goals. First, to induce fatigue within due time to be able to discriminate between a non-fatigued and a fatigued condition in one test. Second, to be able to practically measure the effects of a concurrent attention-demanding task and visual restriction on sprint performance.

Testing physical performance under fatigued conditions has been suggested to improve the possibilities of evaluating the effects of training or rehabilitation [14]. It is also necessary to know if the detected effects are genuine (that is, caused by training or rehabilitation), or if they are caused by the instrumentation system's measurement errors or normal variation of a person's performance. Hence the main purposes of this study were: 1) to assess test-retest reliability of the figure-of-eight sprint test in healthy team-sport athletes, and 2) to study the effect of sprinting with a concurrent attention-demanding task or with restricted visual information on sprint performance in both a non-fatigued and fatigued condition.

## Methods

### Subjects

Healthy team-sport athletes who were physically active at least three times a week and without a history of trauma to the lower extremities and/or other injuries that have been suggested to affect sprinting capabilities were included in the study. The subjects were recruited via sports clubs. Twenty-seven subjects (16 men and 11 women) were included in the study. They had a mean age of 23 ± 3 years, with a mean weight of 74 ± 11 kg and a mean height of 1.81 ± 0.11 m, and they had a median activity level of 85 points (range 75 to 95) according to the Sports Activity Score of the Cincinnati Knee Rating System [15]. A subgroup of 19 subjects (9 men and 10 women) performed the figure-of-eight sprint test twice to determine test-retest reliability. They had a mean age of 22 ± 3 years, with a mean weight of 72 ± 10 kg and a mean height of 1.79 ± 0.11 m, and they had a median activity level of 85 points (range 75 to 95), according to the Sports Activity Score of the Cincinnati Knee Rating System [15]. All subjects gave written informed consent prior to testing. This study and its protocol were approved by the Local Medical Ethics Committee of University Medical Center Groningen.

### Assessment

#### Figure-of-eight sprint test

**Figure 1:**
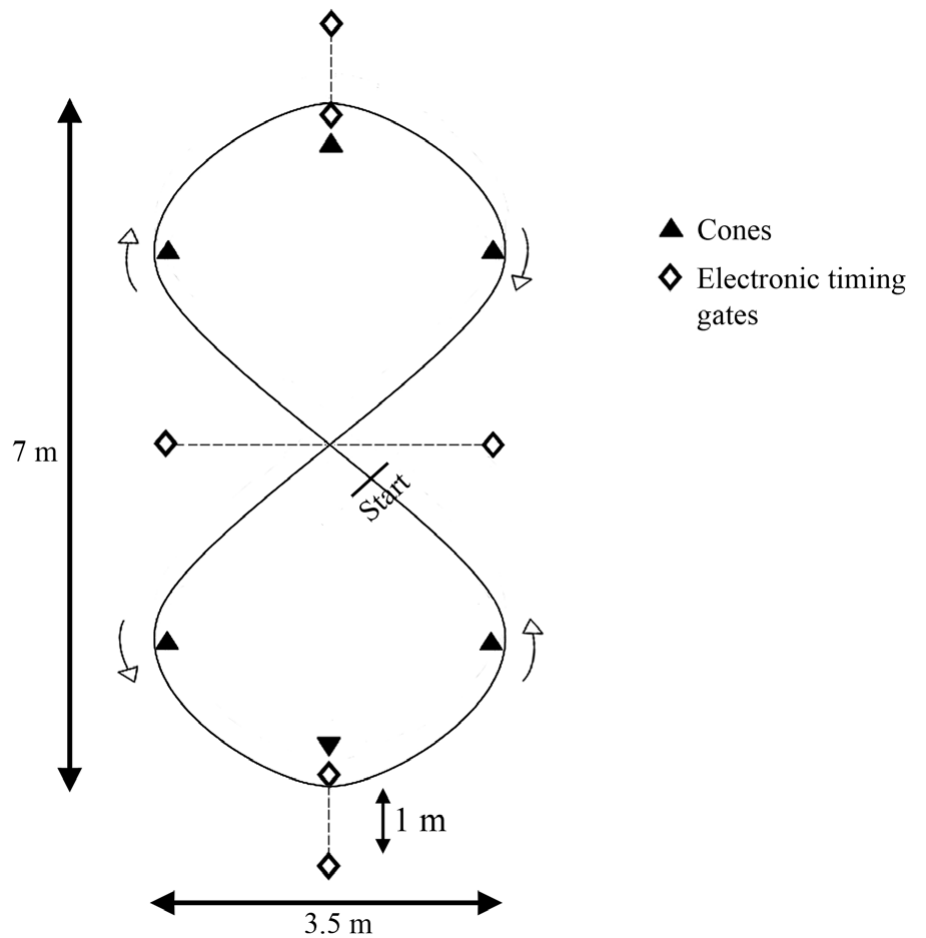
**Figure-of-eight sprint track**.

The sprint track of the sprint test has a figure-of-eight shape (Figure 1), allowing constant directional changes that place continuous strain on the ankle and knee joints. The test has an intermittent character and consists of nine 30-second maximal sprints, with the first three sprints interspersed by a 2-minute active recovery period and a 15-s active recovery period interspersing the subsequent six sprints. After 30 s of maximal exercise, depletion of phosphocreatine (PCr) stores has been reported to be 60-80% from resting values [16]. A recovery period of two minutes after maximal effort is sufficient to restore PCr concentrations up to 90% of resting values [17], reflecting a non-fatigued condition. By shortening the recovery periods to 15 s after the third sprint, the PCr stores can only be partly restored and the oxygen uptake and lactate concentrations remain high [18] resulting in fatigue. From a practical point of view, the sprint duration of 30 s is adequate to measure the effects of restricted vision and attention-demanding task on sprint performance.

Primary outcome measure was the time needed to sprint three laps of the figure-of-eight track per maximal sprint. We chose three laps as the fixed distance because all subjects were able to cover that distance within 30 s. However, the duration of the sprints was set at 30 s, because we wanted to have a consistent work-rest ratio and test duration for all subjects. The first three sprints were interspersed with 2-minute rest periods, to assess sprint performance in a relatively non-fatigued physical condition. The resting period interspersing sprints 4-9 was shortened to 15 s. Subjects were instructed to walk around during these resting periods. The start and finish of each sprint and the subsequent resting period were indicated using sound signals on a pre-recorded compact disc. Sprint times were measured with electronic timing equipment by means of twin-beam photocell gates (HL 2-31 Photocell, Tagheuer, la Chaux-de-Fond, Switzerland), placed approximately 0.75 m above ground. The photocells were linked to an electronic timer with an accuracy of 0.01 s. Each sprint was initiated from a line 30 cm behind the midline of the figure-of-eight, to prevent false triggering of the timing gate.

The test consisted of three parts: sprinting without any restriction (base measurement), sprinting with a concurrent attention-demanding task and sprinting with restricted vision. All test parts had to be conducted on different testing days within two weeks, with at least one day between measurements. The order in which the test parts were conducted was determined by randomisation at the first measurement session.

The amount of attention needed to perform the sprint test was measured by performing an additional attention-demanding task next to the sprint test. This task consisted of an auditory Strooptask in which the subjects heard a man's voice enunciating the words 'high' and 'low' in either a high or a low pitch. Subjects were requested to indicate as fast as possible whether the pitch was high or low, and to suppress the strong tendency to repeat the spoken word [19]. A single 30-second trial of the Strooptask consisted of 12 words. Subjects were instructed to focus on the Strooptask during sprinting. Before starting the sprint test, the Strooptask was first practiced in a sitting position.

Dependency on visual information for sprinting was assessed by means of restricted vision, using goggles with blurry lenses and 15-mm diameter circles right in front of the eyes, thus allowing central vision to remain unlimited and creating restricted (blurry) peripheral vision. The goggles could be worn over corrective spectacles. Lemmink et al. [20] demonstrated that especially the control of directional changes during sprinting decreases when peripheral vision is restricted.

#### Fatigue measurements

Three procedures were used to gain insight into the level of fatigue. First, heart rate was monitored every 5 s during the test with a heart rate transmitter and receiver (Polar S810, Kempele, Finland). After the sprint test, the recorded heart rates were extracted from the receiver onto a personal computer. Mean heart rates per sprint (e.g. without the resting periods) were calculated using software (Polar Precision Performance Software 3.0, Kempele, Finland). To provide an indication of the anaerobic contribution to the sprint test, blood lactate concentrations were obtained from fingertip blood samples before the figure-of-eight sprint test, immediately after the third sprint and after the test (AccuCheck Softclix Pro Lancets, Roche Diagnostics GmbH, Mannheim, Germany), and subsequently analysed for lactate concentrations (YSI 2300 Lactate Analyzer, Yellow Springs, OH, USA). At these three moments Ratings of Perceived Exertion (RPE) were recorded using a 15-point scale [21].

### Procedures

The figure-of-eight sprint test was conducted on a rubberised floor in a sports hall during the subject's normal training hours, varying between 5 and 10 p.m. All test parts were completed at approximately the same time of day. Subjects wore the same shoes during all test parts. Before the warm-up, the RPE scale was explained to the subjects according to the scale instructions [21]. All subjects performed a regular warm-up, consisting of running activities followed by stretching of the leg muscles. Additionally, prior to the regular warm-up subjects were allowed to perform a short general warm-up on a cycle ergometer. All subjects were familiarised with the figure-of-eight track by means of a 30-second practice sprint, and the goggles were used during familiarisation when the test part 'sprinting with restricted vision' had to be performed. To assess test-retest reliability of the figure-of-eight sprint test, the subjects in the subgroup performed all three test parts twice with a 2-week interval between the test and retest sessions.

### Statistical analyses

All statistical analyses were computed using Statistical Package for the Social Sciences (SPSS, Inc., Version 14.0, 2006, Chicago, IL, USA). The level of significance was set at P < 0.05. Measures of centrality and spread are presented as means ± standard deviations. We used the mean sprint time of the first three sprints as mean sprint time in a relatively non-fatigued state, and the mean time of the last three sprints as mean sprint time in a fatigued state. The 4^th^, 5^th ^and 6^th ^sprints were seen as transitional sprints from a non-fatigued to a fatigued physical state. Mean heart rates in non-fatigued condition and fatigued condition consisted of the mean heart rate (without the interspersed resting periods) of the first three sprints and the last three sprints respectively.

#### Test-retest reliability

To gain insight into relative reliability, Intraclass Correlation Coefficients (ICCs) (two-way mixed effects, absolute agreement) and 95% confidence limits (CLs) of the primary outcome measure were calculated [22]. As a general rule, an ICC over 0.90 is considered to be high, between 0.80 and 0.90 moderate, and below 0.80 insufficient for physiological field tests [23]. Baumgartner and Jackson [24] state that ICCs of a minimum of 0.80 are acceptable for physical measures.

The mean difference between test and retest with a 95% CL was calculated for absolute reliability [25]. Zero lying within the 95% CL of the mean difference can be seen as a criterion for absolute reliability. Consequently, when zero lies outside the 95% CL a bias in the measurements is indicated [22,25]. This method was also used to investigate agreement in heart rates, RPE scores and lactate concentrations between test and retest sessions.

#### Effects of attention-demanding task, restricted vision and fatigue on sprint performance

A repeated-measures analysis of variance (ANOVA), with test part as the between-subjects factor and fatigue state as the within-subjects factor, was performed on the sprint times and on the fatigue measurements. Significant main effects for all ANOVA were followed up using Bonferroni adjustments.

## Results

### Test-retest reliability

**Figure 2:**
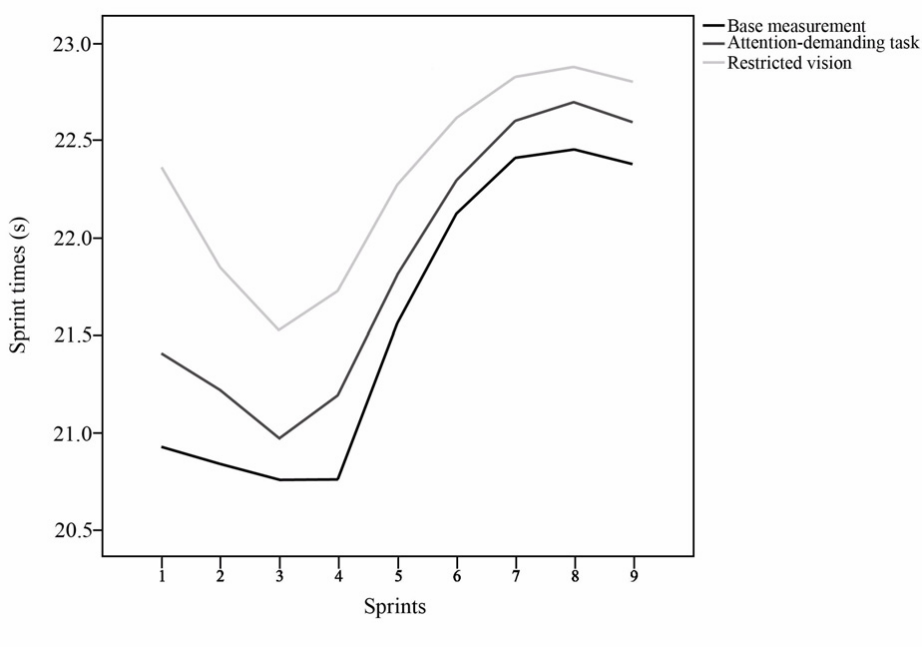
**Sprint times during the first test session of a repeated (9 × 30 s) figure-of-eight sprint test with varying rest periods**. Group data (N = 19) for the sprint times (s) per test part.

The times of the first sprint were higher than those of the subsequent sprints (Figure 2). The athletes probably had a subconscious tendency to hold back for later sprints. Furthermore, when the first sprint was excluded from the sprint time in a non-fatigued state the ICCs increased (Table 1), therefore we excluded the first sprint from further analysis and used the mean sprint time of the 2^nd ^and 3^rd ^sprints as mean sprint time in a relatively non-fatigued state. Conversely, the 9^th ^and last sprint was faster than the previous sprints, probably because the subjects gave all they had left for their final sprint performance. The ICCs also increased when the last sprint was excluded from the sprint time in a fatigued state (Table 1), hence we used the mean time of the 7^th ^and 8^th ^sprints as mean sprint time in a fatigued state. Consequently, the mean heart rates in non-fatigued state and fatigued state consisted of the mean heart rate (without the interspersed resting periods) of the 2^nd ^and 3^rd ^sprints, and the 7^th ^and 8^th ^sprints respectively.

**Table 1:** Intraclass correlation coefficients for relative reliability of sprint times during a repeated (9 × 30 s) figure-of-eight sprint test with varying rest periods with and without the first and last sprints.*†

		Base measurement	Attention-demanding task	Restricted vision
**NFC**	First sprint included	0.77 (0.50 - 0.90)	0.78 (0.54 - 0.92)	0.85 (0.67 - 0.93)
	First sprint excluded	0.80 (0.60 - 0.92)	0.83 (0.61 - 0.93)	0.86 (0.67 - 0.94)
**FC**	Last sprint included	0.90 (0.74 - 0.96)	0.82 (0.59 - 0.92)	0.68 (0.35 - 0.86)
	Last sprint excluded	0.94 (0.85 - 0.98)	0.85 (0.65 - 0.94)	0.75 (0.45 - 0.89)

* NFC, non-fatigued condition; FC, fatigued condition.

† Values in parentheses are 95% confidence limits.

The figure-of-eight sprint test revealed a good level of test-retest reliability as evidenced by ICC values ranging from 0.75 for the test part 'restricted vision fatigued' to 0.94 for the test part 'base measurement fatigued' (95% likely range: 0.40-0.98), and by the values of the mean differences between test and retest that were small when compared with the means of test and retest with zero lying within the 95% CL of all test parts (Table 2). Agreement data for heart rates, RPE scores and lactate concentrations are presented in Table 3. Zero lay within the 95% CL of all fatigue measurements.

**Table 2:** Absolute reliability data for sprint times during a repeated (9 × 30 s) figure-of-eight sprint test with varying rest periods.*†‡

		Test	Retest	Mean difference	95% CL
**Base measurement**	**NFC**	20.8 (1.0)	21.0 (1.0)	-0.2	-0.5 - 0.1
	**FC**	22.4 (1.2)	22.3 (1.4)	0.1	-0.1 - 0.3
**Attention-demanding task**	**NFC**	21.1 (1.1)	21.0 (1.1)	0.1	-0.2 - 0.4
	**FC**	22.7 (1.2)	22.5 (1.3)	0.2	-0.1 - 0.5
**Restricted vision**	**NFC**	21.7 (1.3)	21.6 (1.2)	0.1	-0.2 - 0.4
	**FC**	22.9 (1.3)	22.6 (1.4)	0.2	-0.2 - 0.7

*95% CL, 95% confidence limits; NFC, non-fatigued condition; FC, fatigued condition.

^† ^All times are expressed in seconds.

^‡ ^Values in parentheses are standard deviations.

**Table 3:** Agreement data for heart rates, RPE scores and lactate concentrations obtained during a repeated (9 × 30 s) figure-of-eight sprint test with varying rest periods.* †‡

		Test	Retest	Mean difference	95% CL
**Heart rates**					
Base measurement	**P**	76.9 (10.9)	79.9 (15.5)	-3.0	-13.4 - 7.4
	**NFC**	151.6 (10.1)	144.5 (16.9)	7.1	-0.4 - 14.5
	**FC**	186.9 (7.2)	184.1 (9.9)	2.8	-0.3 - 5.9
Attention-demanding task	**P**	80.3 (17.2)	78.8 (10.9)	1.5	-8.5 - 11.5
	**NFC**	145.6 (3.8)	142.8 (14.9)	2.8	-1.5 - 7.1
	**FC**	186.4 (9.2)	184.6 (8.4)	1.7	-1.0 - 4.4
Restricted vision	**P**	78.5 (11.4)	79.6 (9.9)	-1.1	-10.3 - 7.8
	**NFC**	148.2 (13.5)	141.3 (18.3)	6.9	-0.3 - 14.2
	**FC**	185.1 (7.6)	183.6 (8.1)	1.5	-0.2 - 3.1
**RPE scores**					
Base measurement	**P**	8.3 (2.1)	8.0 (2.3)	0.3	-1.0 - 1.6
	**NFC**	13.2 (1.7)	12.6 (1.6)	0.6	-0.3 - 1.5
	**FC**	16.7 (1.6)	16.5 (1.4)	0.2	-0.2 - 0.6
Attention-demanding task	**P**	8.0 (2.4)	8.1 (2.3)	-0.1	-1.1 - 0.9
	**NFC**	11.8 (1.5)	12.1 (1.9)	-0.2	-1.4 - 1.0
	**FC**	15.2 (1.5)	15.8 (1.7)	-0.7	-1.6 - 0.2
Restricted vision	**P**	7.9 (2.1)	8.3 (2.4)	-0.4	-1.1 - 0.3
	**NFC**	12.5 (1.8)	12.6 (1.9)	-0.1	-1.0 - 0.9
	**FC**	15.7 (1.7)	16.2 (1.3)	-0.5	-1.3 - 0.3
**Lactate concentrations**					
Base measurement	**P**	2.1 (1.3)	1.7 (0.6)	0.2	-0.1 - 0.4
	**NFC**	7.3 (1.8)	6.6 (2.5)	0.4	-0.4 - 1.1
	**FC**	10.3 (2.7)	9.8 (2.2)	0.2	-0.0 - 0.6
Attention-demanding task	**P**	1.9 (0.9)	1.9 (1.0)	-0.0	-0.3 - 0.3
	**NFC**	6.4 (2.0)	6.1 (1.5)	0.2	-0.4 - 0.7
	**FC**	9.5 (3.8)	8.4 (2.1)	0.6	-3.4 - 4.5
Restricted vision	**P**	1.8 (0.5)	1.5 (0.4)	0.1	-0.0 - 0.3
	**NFC**	6.1 (2.1)	5.8 (1.3)	0.2	-0.3 - 0.7
	**FC**	9.4 (2.8)	8.8 (1.8)	0.3	-0.1- 0.7

* 95% CL, 95% confidence limits; P, pre-test assessment; NFC, assessment at sprint 3; FC, assessment at the end of the sprint test.

^† ^Heart rates are expressed in beats·min^-1 ^and lactate concentrations as mmol·L^-1^.

^‡ ^Values in parentheses are standard deviations.

### Effects of attention-demanding task, restricted vision and fatigue on sprint performance

As in the test-retest reliability analysis, the first and last sprints were excluded from further analysis. Sprint times of the figure-of-eight sprint test are presented in Table 4. There was a significant main effect of test part on sprint times (P < 0.001). Post-hoc analyses revealed that the sprint times on the test part 'base measurement' were significantly lower than those on the test parts 'attention-demanding task' (P = 0.01) and 'restricted vision' (P < 0.001), regardless of fatigue state. There was also a significant main effect of fatigue state on sprint times (P < 0.001), indicating a significant increase in sprint times in fatigued condition on all test parts, and there was a significant interaction effect between test part and fatigue state (P = 0.03).

**Table 4:** Mean sprint times during a repeated (9 × 30 s) figure-of-eight sprint test with varying rest periods.* †‡

	Base measurement	Attention-demanding task	Restricted vision
**NFC**	20.7 (0.8)	21.0 (1.0)	21.4 (1.0)
**FC**	22.0 (1.1)	22.4 (1.1)	22.6 (1.1)

* NFC, non-fatigued condition; FC, fatigued condition.

^† ^Sprint times are expressed in seconds.

^‡ ^Values in parentheses are standard deviations.

Table 5 shows the mean and standard deviations of the heart rates, RPE scores and lactate concentrations. There was no significant main effect of test part on heart rates, lactate concentrations or RPE scores (P > 0.05), indicating comparable heart rates, RPE scores and lactate concentrations on all test parts. The main effect of fatigue state was significant (P < 0.001) for all fatigue measurements, indicating a significant increase in heart rates, RPE scores and lactate concentrations in fatigued condition on all test parts.

**Table 5:** Heart rates, RPE scores and lactate concentrations obtained during a repeated (9 × 30 s) figure-of-eight sprint test with varying rest periods.* †‡

		Heart rate	RPE scores	Lactate concentrations
**Base measurement**	**P**		7.3 (1.3)	1.9 (0.9)
	**NF**	149.6 (12.7)	12.1 (1.8)	6.8 (1.6)
	**F**	187.7 (8.4)	16.3 (1.8)	9.8 (2.6)
**Attention-demanding task**	**P**		7.3 (1.4)	1.9 (0.7)
	**NF**	145.7 (16.2)	11.2 (1.4)	6.3 (1.5)
	**F**	183.7 (8.8)	15.3 (1.7)	8.9 (2.5)
**Restricted vision**	**P**		7.5 (1.9)	1.9 (0.7)
	**NF**	146.3 (15.4)	11.4 (1.8)	6.0 (1.4)
	**F**	182.9 (8.4)	16.0 (1.4)	8.4 (2.3)

* 95% CL, 95% confidence limits; P, pre-test assessment; NF, assessment at sprint 3; F, assessment at the end of the sprint test.

^† ^Heart rates are expressed in beats·min^-1 ^and lactate concentrations as mmol·L^-1^.

^‡ ^Values in parentheses are standard deviations.

## Discussion

Using two basic characteristics of field-based team sports, namely repeated sprint and multidirectional nature, we developed a figure-of-eight sprint test. This test allows insight into not only the physical condition but also the attentional and visual demands for sensorimotor control. Overall, the figure-of-eight sprint test showed good test-retest reliability. Furthermore, the attentional and visual demands for sprint performance, in both non-fatigued and fatigued condition, can be measured in healthy team-sport athletes by means of the figure-of-eight sprint test.

To determine test-retest reliability, ICC values, mean difference and 95% CL of the mean differences have been reported as appropriate and clear, though several authors use other measures (such as Pearson's correlation coefficient, coefficient of repeatability and coefficient of variation) [22]. As a general rule, ICCs of a minimum of 0.80 are considered acceptable for physical measures [23,24]. All test parts of the figure-of-eight sprint test, except for 'restricted vision fatigued', met the 0.80 criterion for relative reliability. Between-subject variation was relatively high in the test part 'restricted vision fatigued', which is known to influence the magnitude of the ICC value [22]. Conversely, absolute reliability of this test part is proven to be acceptable. Absolute reliability of the figure-of-eight sprint test is supported by the finding that the values of the mean differences for the sprint times of all test parts were small when compared with the means at both test and retest, and that zero was within all 95% CLs.

There are two components of variability associated with each assessment of measurement error: systematic bias (i.e. changes in a measure over time such as learning effects) and random error due to inherent subject or instrument variation [26]. Learning effects may occur when subjects have not had experience with or practice at the test before being measured. Random errors are often due to changes in measurement equipment, location of the measurements and changes in the subjects. The measurement protocol used in this study comprised familiarisation sprints, both of the sprint track and of the visual restriction and attention-demanding task, to control for learning effects. Furthermore, tests were conducted in the same sports hall at approximately the same time of day and subjects wore the same shoes during the test and retest sessions.

The reliability coefficients of this study are in line with those obtained when evaluating other sprint tests. Boddington et al. [27] investigated the test-retest reliability of a modified 5-m multiple shuttle test to determine the 'match-related fitness' of 23 female hockey players. They reported a range in ICCs of 0.74-0.98. A study to determine the test-retest reliability of a shuttle sprint and dribble test for field hockey players reported ICCs ranging from 0.71 to 0.91, and zero lay within the 95% CL [28]. Lemmink et al. [29] reported ICCs of 0.86 and higher, with zero lying within the 95% CL of an interval shuttle-run test in 17 intermittent sport players. They also conducted RPE scores and heart rate measurements, which have shown to be consistent over time. A study to determine the test-retest reliability of a multiple sprint running performance test by 11 physically active men showed ICCs ranging from 0.79 to 0.94 [30].

All indicators of fatigue showed that fatigue was created. In general, the values of the mean differences in heart rates were small when compared with the means at test and retest. Borg's RPE scores were also consistent over time; mean differences of RPE scores were small (less than 1 point difference on the RPE scale). Mean differences in lactate concentrations between test and retest were small, ranging between 0.02 and 0.55 mmol L^-1^. With zero included in the 95% CL of all test parts, it can be concluded that heart rates, lactate concentrations and RPE scores were consistent between both test sessions, indicating that the amount of fatigue created during test and retest was comparable.

The second aim of this study was to examine the effect of sprinting with a concurrent attention-demanding task or with restricted vision on sprint performance in both non-fatigued and fatigued condition. Physical testing in a fatigued physical condition may provide valuable information, due to which deficiencies in an individual's physical-functional capabilities may appear unnoticed when testing only in a non-fatigued condition. Fatigue was induced by shortening the resting period between sprints. A significant decline in sprint times was seen when the resting periods were shortened, irrespective of the test part. All fatigue measurements increased significantly in fatigued condition and indicated a maximal or near-maximal effort on all test parts of the sprint test. Heart rates in fatigued condition exceeded 90% of the predicted maximal heart rate (maximal heart rate (beats min^-1^) = 220 - age (years) [31]) for all test parts. Lactate concentrations at the end of all test parts exceeded 8 mmol L^-1^. RPE conducted at the end of the figure-of-eight sprint test were higher than 15 (heavy exertion) for all test parts.

Sprint times increased significantly when the sprint test was performed with an attention-demanding task or with restricted vision irrespectively of level of fatigue. This indicates that, regardless of whether the athletes were fatigued, sprinting required attention and visual information. This is an important finding, since it shows that even healthy athletes could not perform the sprints automatically, and that they were dependent on visual information for sprinting. Peripheral vision provides valuable information for the control of one's own movements, thereby playing an important role in sensorimotor control. However, sports matches are dynamic situations in which athletes should be focused on activities in their environment and not on their own movements. Sports training enhances the use of somatosensory information and decreases dependency on attention and visual information. Paillard et al. [32] investigated whether level of competition in soccer influences postural performance. They found that professional soccer players were less dependent on visual information for postural control than regional-level soccer players. They are more skilled at using other sources of input (such as proprioceptive information) to keep motor performance optimal. This underlines the notion that not only the physical condition of athletes should be monitored, but also their attentional and visual demands for sensorimotor control. Moreover, when there is loss of proprioceptive input from the lower extremities due to damage of the musculoskeletal system, the attentional and visual demands of sensorimotor control are higher compared to healthy subjects. De Visser et al. [33] and Okuda et al. [34] reported that the attentional and visual demands of gait and postural control were raised in patients after limb-saving surgery and after ACL injury.

Additionally, the test part-level of fatigue interaction was significant, illustrating that the attentional and visual demands of sprinting increased in a fatigued physical state. Research has shown that attention and visual information can largely compensate for a disturbed proprioceptive feedback due to fatigue [9,10]. This demonstrates that even healthy athletes are more susceptible to injuries when fatigued. More significantly, it can be proposed that people with impaired sensorimotor control (e.g. athletes with ankle sprains or with ACL injuries) whose attentional and visual demands are already raised in a non-fatigued physical condition are disproportionately dependent on attention and visual information when fatigued, which places them at a higher risk for re-injury.

## Conclusions

High ICCs and the absence of systematic variation indicate a satisfactory relative and absolute reliability of the figure-of-eight sprint test. The attentional and visual demands for sprinting of healthy athletes can be assessed with the figure-of-eight sprint test. Fatigue resulted not only in a significantly decreased sprint performance, it also increased attentional and visual demands for sprinting. Further research is needed to gain insight into the applicability of the test in a sports medicine setting to assess the attentional and visual demands for sprint performance in athletes undergoing rehabilitation after injuries to the lower extremities, such as knee injuries.

## Competing interests

The authors declare that they have no competing interests.

## Authors' contributions

IHFR participated in the design of the study and in collecting the data, performed the statistical analysis and drafted the manuscript. KAPML, RLD and MS participated in the design of the study and in the progress and revision of the manuscript. ATB participated in collecting the data and drafting the manuscript. All authors read and approved the final manuscript.

## Pre-publication history

The pre-publication history for this paper can be accessed here:

http://www.biomedcentral.com/1471-2474/11/84/prepub
